# A proposed GABAergic pathway in the feeding neural network is associated with learning performance in conditioned taste aversion in *Lymnaea*

**DOI:** 10.1242/bio.062762

**Published:** 2026-10-16

**Authors:** Ayaka Itoh, Samui Chiba, Yoshimasa Komatsuzaki, Minoru Saito

**Affiliations:** ^1^ Department of Correlative Study in Physics and Chemistry, Graduate School of Integrated Basic Sciences, Nihon University, 3-25-40 Sakurajosui, Setagaya-ku, Tokyo 156-8550, Japan; ^2^ Department of Physics, Graduate School of Science and Technology, Nihon University, 1-8-14 Kandasurugadai, Chiyoda-ku, Tokyo 101-8308, Japan

**Keywords:** Conditioned taste aversion, Long-term memory, GABAergic signaling, Cerebral giant cell, Learning performance, *Lymnaea stagnalis*

## Abstract

Conditioned taste aversion (CTA) provides a useful model for investigating the neural mechanisms underlying long-term memory (LTM). In *Lymnaea stagnalis*, CTA can be induced by pairing sucrose, which initially elicits feeding responses, with KCl, which suppresses feeding responses. Following such conditioning, snails exhibit a long-lasting reduction in feeding responses to sucrose, reflecting the formation of CTA-LTM. In the present study, however, individual snails exhibited marked variability in CTA-LTM performance. We therefore analyzed behavioral differences among learner phenotypes and examined how pharmacological manipulation of GABAergic signaling during CTA training influences learning performance. Behavioral analyses revealed that learner phenotypes were associated with differences in feeding responses to sucrose before training. External application of the GABA_A_ receptor antagonist bicuculline significantly increased the proportion of good learners. Based on the present findings, together with previous results, we propose a working model incorporating a putative GABAergic pathway within the feeding neural network, possibly involving the cerebral giant cell, and suggest that this pathway may be associated with individual differences in learning performance. Although the precise neural mechanisms remain to be determined, the present study provides a framework for understanding how GABAergic signaling contributes to CTA-LTM formation and individual variation in learning performance.

## Teaser

**Summary:** Behavioral and pharmacological analyses suggest that GABAergic regulation within the feeding neural network contributes to individual differences in conditioned taste aversion learning in *Lymnaea*.

## INTRODUCTION

Learning and memory allow animals to modify their behavior according to past experience and environmental conditions. A central question in neuroscience is how neural circuits convert sensory experience into persistent behavioral changes. Conditioned taste aversion (CTA) is a well-established form of associative learning in which an initially appetitive stimulus becomes aversive after being paired with an unpleasant stimulus. CTA is widely used to investigate the neural and molecular mechanisms underlying long-term memory (LTM). The mechanisms of CTA have been extensively studied in both vertebrate and invertebrate systems. Among invertebrate systems, the pond snail *Lymnaea stagnalis* has been used as an important model organism (Benjamin et al., 2000; Benjamin, 2012). Its central nervous system (CNS) consists of a relatively small number of large and identifiable neurons, which makes it possible to analyze the neural circuitry underlying behavior at the level of individual neurons (Benjamin and Elliott, 1989; Kemenes and Elliott, 1994). In *Lymnaea*, CTA can be experimentally induced by pairing sucrose [conditioned stimulus (CS)], which initially elicits feeding responses, with KCl [unconditioned stimulus (UCS)], which suppresses feeding responses. Following such conditioning, the snails exhibit a long-lasting reduction in their feeding responses to sucrose, reflecting the formation of CTA-LTM (Kojima et al., 1996, 1997; Yamanaka et al., 1999; Sugai et al., 2007; Ito et al., 2012, 2015). However, individual snails exhibit marked variability of learning performance in CTA-LTM formation.

Feeding behavior in *Lymnaea* is generated by a well-characterized neural circuit that includes a central pattern generator (CPG) located in the buccal ganglia. The activity of this feeding circuit is strongly influenced by modulatory neurons located in the cerebral ganglia. Among these neurons, the cerebral giant cell (CGC) plays a central role in regulating feeding behavior. The CGC modulates the excitability of the feeding CPG and contributes to the initiation and maintenance of feeding motor patterns (McCrohan and Audesirk, 1987; Yeoman et al., 1996; Benjamin, 2012). Previous studies have also demonstrated that the CGC is essential in CTA-LTM formation (Kojima et al., 1997; Ito et al., 2012).

GABA is the principal inhibitory neurotransmitter in both vertebrate and invertebrate nervous systems and plays important roles in regulating neuronal excitability and synaptic plasticity associated with memory formation (Isaacson and Scanziani, 2011; Caroni, 2015; Letzkus et al., 2015; Ferguson and Cardin, 2020). In *Lymnaea*, GABAergic neurons are widely distributed throughout the CNS (Hatakeyama and Ito, 2000), and GABAergic transmission modulates the activity of feeding-related neurons (Elliott and Kemenes, 1992; Yeoman et al., 1996). Our previous electrophysiological study demonstrated that bath application of GABA to isolated CNS preparations suppresses the spontaneous firing activity of the CGC after CTA-LTM formation (Itoh et al., 2021). In addition, epicatechin, a flavonoid that has been reported to affect memory-related processes in animals (van Praag et al., 2007; Spencer, 2009; Fruson et al., 2012), also suppresses CGC excitability similarly to GABA and prolongs the persistence of CTA-LTM (Itoh et al., 2021).

Despite these previous results, the contribution of GABAergic signaling within the feeding neural network to CTA-LTM formation and the variability in learning performance among individual snails remain unclear. In the present study, we analyzed behavioral differences among learner phenotypes and examined the effects of pharmacological manipulation of GABAergic signaling during CTA training on learning performance. Based on the obtained behavioral findings, together with previous results, we propose a working model and discuss possible mechanisms underlying individual variability in CTA-LTM formation. In addition, the effect of epicatechin on CTA-LTM formation is also discussed. The proposed working model is hypothetical but expected to guide future experimental studies.

## RESULTS

Fig. 1A shows the proportions of good, poor+ and poor− learners in the distilled water (DW)-trained group (*n*=80), which were 46.3%, 22.5% and 31.2%, respectively. According to our previous definition (Itoh et al., 2021), these data correspond to 46.2% of good learners and 53.8% of poor learners. Here, these proportions of good and poor learners were different from those reported in our previous study (good learners: 67%, poor learners: 33%), as mentioned above. We will discuss this difference in the Discussion. Fig. 1C shows individual rates of decrease in the number of bites at the 24 h posttest compared to that at the pretest in the DW-trained group.

**Fig. 1.**
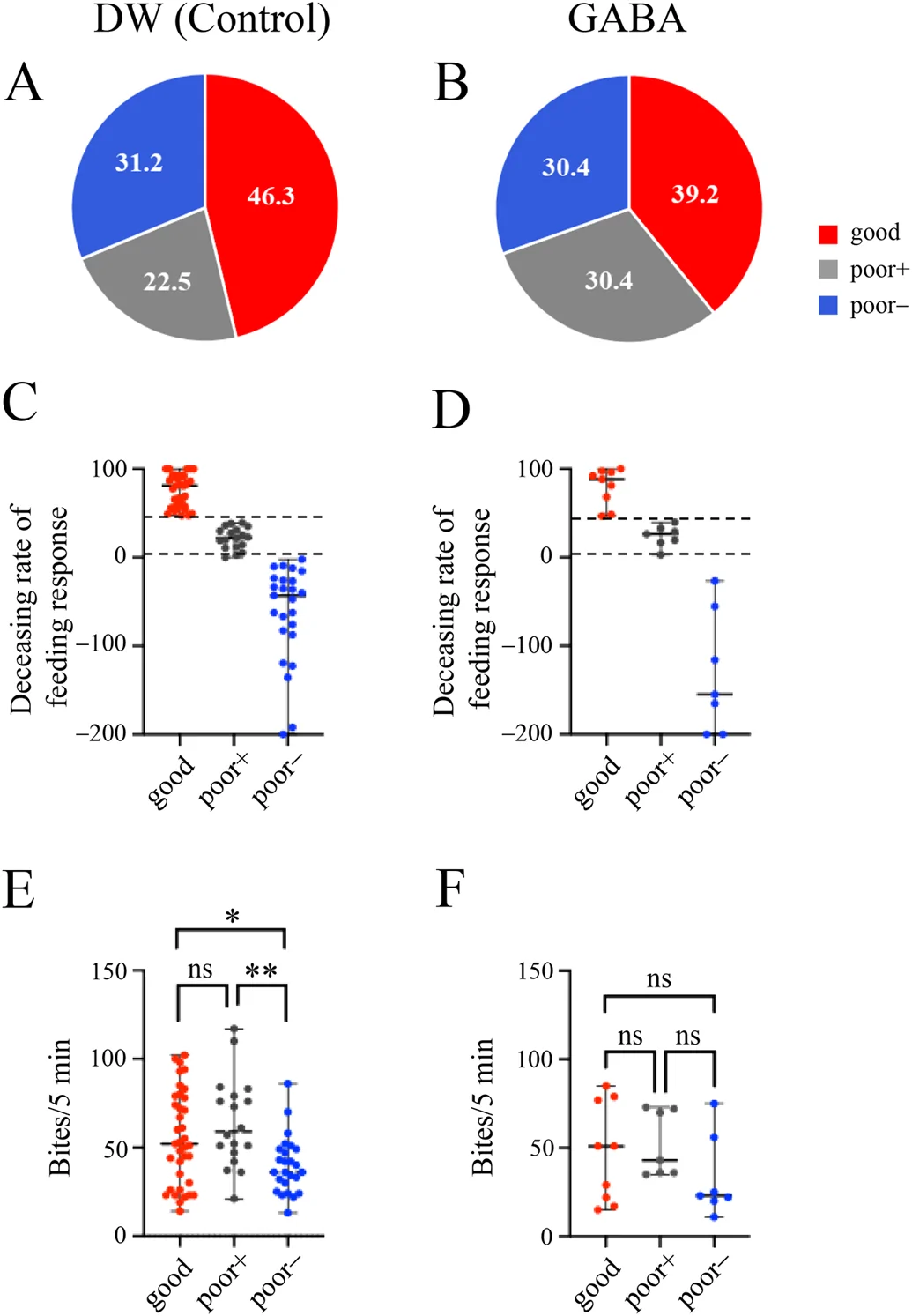
**Effects of GABA treatment during training on learner phenotype distribution, decreasing rates of feeding response to sucrose, and intrinsic feeding responses to sucrose.** (A,B) Proportions of good, poor+ and poor− learners in (A) the DW-trained group (control) (*n*=80) and (B) the GABA-trained group (*n*=23). (C,D) Rates of decrease in the number of bites during 5 min at the 24 h posttest compared to that at the pretest in (C) the DW-trained group (control) (good learners: *n*=37, poor+ learners: *n*=18, poor− learners: *n*=25) and (D) the GABA-trained group (good learners: *n*=9, poor+ learners: *n*=7, poor− learners: *n*=7). The upper dashed lines indicate 40%, which separates the good and poor+ learner phenotypes, and the lower dashed lines show 0%, which separates the poor+ and poor− learner phenotypes. Data are presented as individual values and medians; maximum lines are also indicated, whereas minimum lines are not shown because some values are below −200%. The values are −277% in C and −286% and −672% in D. These data are plotted on the −200% lines. (E,F) The initial number of bites for 5 min at the pretest in (E) the DW-trained group (control) and (F) the GABA-trained group. Data are presented as individual values, medians and ranges (minimum-maximum). Each individual value in C-F represents an individual snail (biological replicate). The experiments were performed in 50 independent experimental sessions for the DW-trained group and 18 independent experimental sessions for the GABA-trained group. The proportions of learner phenotypes in A and B were compared using Fisher's exact test for a 2×3 contingency table. Data in E and F were compared using the Kruskal-Wallis test followed by Dunn's test with the Holm-Bonferroni correction for multiple comparisons. Where applicable, statistical tests were two sided. *P<0.05, **P<0.01, ns: not significant.

To distinguish the good, poor+ and poor− learner phenotypes, we focused on their intrinsic feeding responses to the appetitive solution (10 mM sucrose; CS), that is, their initial numbers of bites at the pretest. They were 52 (14-102) (bites/5 min) for good learners (*n*=37), 59 (21-117) (bites/5 min) for poor+ learners (*n*=18) and 36 (13-86) (bites/5 min) for poor− learners (*n*=25) (Fig. 1E). The initial (i.e. at the pretest) number of bites of poor− learners was significantly lower than that of good learners (Dunn's test with the Holm-Bonferroni correction, *P*=0.036) or poor+ learners (Dunn's test with the Holm-Bonferroni correction, *P*=0.006). In addition, the initial number of bites of poor+ learners was slightly higher than that of the good learners, though no significant difference was observed between the two groups (Dunn's test with the Holm-Bonferroni correction, *P*=0.225). Thus, the differences among the good, poor+ and poor− learner phenotypes reflect their intrinsic feeding responses to the appetitive solution.

The CGC has been shown to both modulate the neural network underlying feeding behavior (McCrohan and Audesirk, 1987; Yeoman et al., 1996; Benjamin, 2012) and be necessary for CTA-LTM formation (Kojima et al., 1997; Ito et al., 2012; Sunada et al., 2017a,b). Our previous study showed that the firing rate of the CGC was reduced by bath application of GABA to isolated CNS preparations of good learners, suggesting that GABAergic inhibition may influence the neural dynamics underlying CTA learning (Itoh et al., 2021). We therefore performed CTA training using GABA solution instead of DW after the five conditioning trials (sucrose followed by KCl). The proportions of good, poor+ and poor− learners in the GABA-trained group (*n*=23) were 39.2%, 30.4% and 30.4%, respectively (Fig. 1B). These data were similar to those in the control (DW-trained group; Fig. 1A) (Fisher's exact test for a 2×3 contingency table with the Holm-Bonferroni correction, *P*=1.000). Fig. 1D shows individual rates of decrease in the number of bites at the 24 h posttest compared to that at the pretest. The initial numbers of bites were 51 (15-85) (bites/5 min) for good learners (*n*=9), 43 (35-73) (bites/5 min) for poor+ learners (*n*=7) and 23 (11-75) (bites/5 min) for poor− learners (*n*=7) (Fig. 1F). No significant differences were seen among the three learner phenotypes (Kruskal-Wallis test, *P*=0.290).

We also performed CTA training using epicatechin solution instead of DW to examine whether epicatechin affected learner phenotype distribution in addition to its previously reported effect on CTA-LTM persistence (Itoh et al., 2021). The proportions of good, poor+ and poor− learners in the epicatechin-trained group (*n*=52) were 40.4%, 21.2% and 38.5%, respectively (Fig. S1A). These data were similar to those in the control (DW-trained group; Fig. 1A) (Fisher's exact test for a 2×3 contingency table with the Holm-Bonferroni correction, *P*=1.000). The initial numbers of bites were 49 (13-94) (bites/5 min) for good learners (*n*=21), 64 (32-86) (bites/5 min) for poor+ learners (*n*=11) and 34.5 (19-71) (bites/5 min) for poor− learners (*n*=20) (Fig. S1B).

Next, we performed CTA training using bicuculline (an antagonist for the GABA_A_ receptor) solution. Here, the DMSO-trained group was used as the control, as mentioned in the Materials and Methods. The proportions of the good, poor+ and poor− learners in the DMSO-trained group (*n*=43) were 41.9%, 30.2% and 27.9%, respectively (Fig. 2A). Fig. 2C shows individual rates of decrease in the number of bites at the 24 h posttest compared to that at the pretest. The initial numbers of bites were 56.5 (13-102) (bites/5 min) for good learners (*n*=18), 74 (12-112) (bites/5 min) for poor+ learners (*n*=13) and 33.5 (14-70) (bites/5 min) for poor− learners (*n*=12) (Fig. 2E). Similarly to the DW-trained group, the initial number of bites of poor− learners was significantly lower than that of poor+ learners (Dunn's test with the Holm-Bonferroni correction, *P*=0.003), and it was also much lower than that of good learners, though no significance was observed between the two groups (Dunn's test with the Holm-Bonferroni correction, *P*=0.114).

**Fig. 2.**
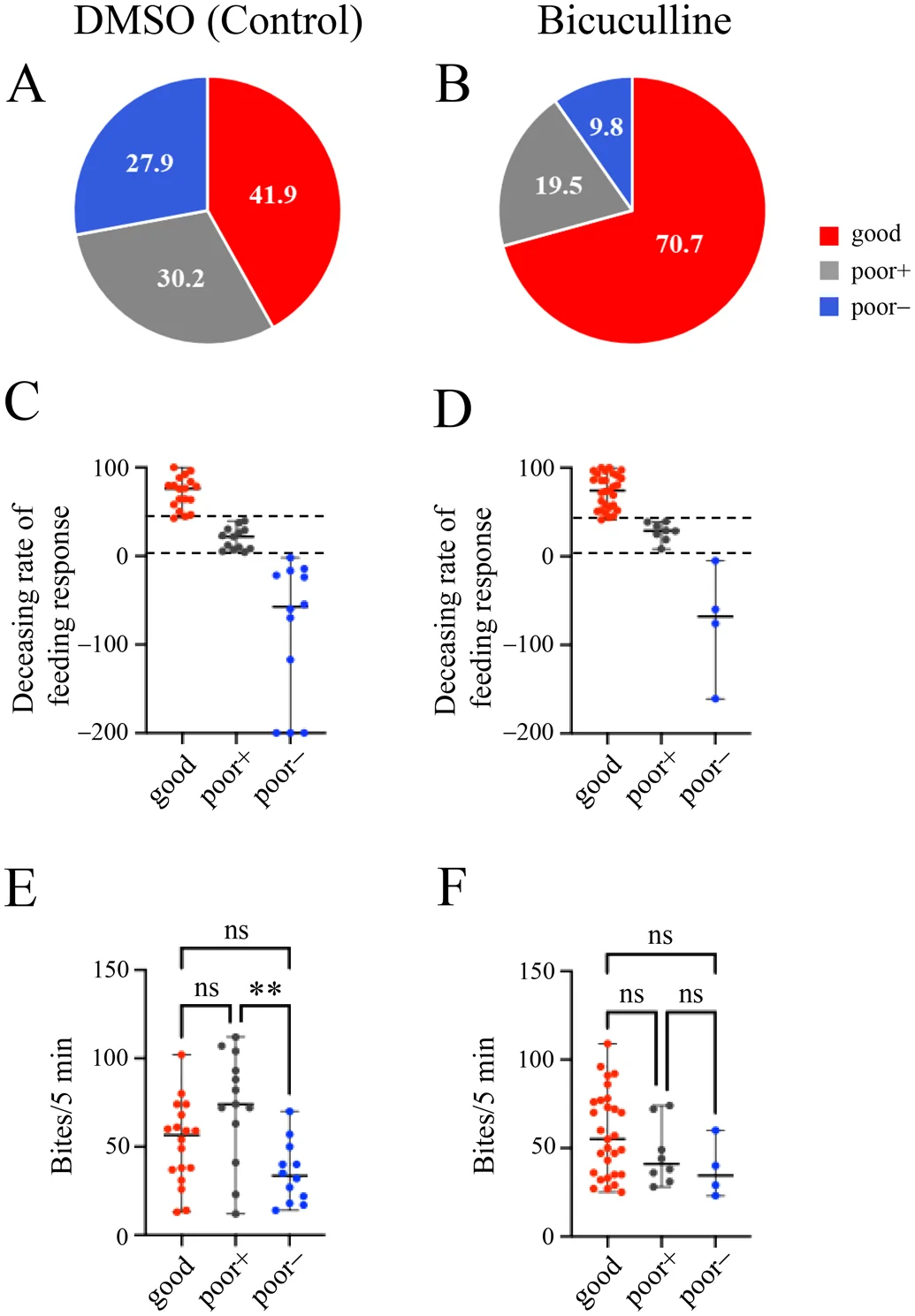
**Effects of bicuculline treatment during training on learner phenotype distribution, decreasing rates of feeding response to sucrose, and intrinsic feeding responses to sucrose.** (A,B) Proportions of good, poor+ and poor− learners in (A) the DMSO-trained group (control) (*n*=43) and (B) the bicuculline-trained group (*n*=41). (C,D) Rates of decrease in the number of bites during 5 min at the 24 h posttest compared to that at the pretest in (C) the DMSO-trained group (control) (good learners: *n*=18, poor+ learners: *n*=13, poor− learners: *n*=12) and (D) the bicuculline-trained group (good learners: *n*=29, poor+ learners: *n*=8, poor− learners: *n*=4). The upper dashed lines indicate 40%, which separates the good and poor+ learner phenotypes, and the lower dashed lines indicate 0%, which separates the poor+ and poor− learner phenotypes. Data are presented as individual values and medians; maximum lines are also indicated, whereas the minimum line in C is not shown because three values are −200% or below. The three values are −200%, −274% and −341%. These data are plotted on the −200% line in C. (E,F) The initial number of bites during 5 min at the pretest in (E) the DMSO-trained group (control) and (F) the bicuculline-trained group. Data are presented as individual values, medians and ranges (minimum-maximum). Each individual value in C-F represents an individual snail (biological replicate). The experiments were performed in 18 independent experimental sessions for the DMSO-trained group and 26 independent experimental sessions for the bicuculline-trained group. The proportions of learner phenotypes in A and B were compared using Fisher's exact test for a 2×3 contingency table. Data in E and F were compared using the Kruskal-Wallis test followed by Dunn's test with the Holm-Bonferroni correction for multiple comparisons. Where applicable, statistical tests were two sided. **P<0.01, ns: not significant.

The proportions of good, poor+ and poor− learners in the bicuculline-trained group (*n*=41) were 70.7%, 19.5% and 9.8%, respectively (Fig. 2B). These data were significantly different from those of the control (DMSO-trained group; Fig. 2A) (Fisher's exact test for a 2×3 contingency table, *P*=0.022), in which the proportion of good learners became much higher in the bicuculline-trained group than in the control. Fig. 2D shows individual rates of decrease in the number of bites at the 24 h posttest compared to that at the pretest. The initial (i.e. at the pretest) numbers of bites were 55 (25-109) (bites/5 min) for good learners (*n*=29), 41 (28-74) (bites/5 min) for poor+ learners (*n*=8) and 34.5 (23-60) (bites/5 min) for poor− learners (*n*=4) (Fig. 2F). No significant differences were seen among the three learner phenotypes (Kruskal-Wallis test, *P*=0.193).

Fig. 3 shows the associations between intrinsic feeding responses to sucrose and learner phenotype probabilities in the DMSO-trained group using logistic regression analyses. From the logistic regression curve of good versus poor+ learners, the probability that snails are classified in the poor+ learner phenotype is higher than 70% when the initial number of bites is higher than 100. Therefore, the upper group of good learners in the bicuculline-trained group shown in Fig. 2F could be ‘intrinsically’ classified into the poor+ learner phenotype. Similarly, from the logistic regression curve of poor+ versus poor− learners, the probability that snails are classified into the poor− learner phenotype is higher than 70% when the initial number of bites is lower than 40. Therefore, the lower group of poor+ learners in the bicuculline-trained group shown in Fig. 2F could be ‘intrinsically’ classified into the poor− learner phenotype. These shifts may lead to the lower initial number of bites of poor+ learners compared with the control (DMSO-trained group), resulting in the disappearance of the statistical significance between the initial number of bites of poor+ learners and that of poor− learners. If this speculation is correct, the proportions of the ‘intrinsic’ good, poor+ and poor− learners in the bicuculline-trained group could be closer to those of the control.

**Fig. 3.**
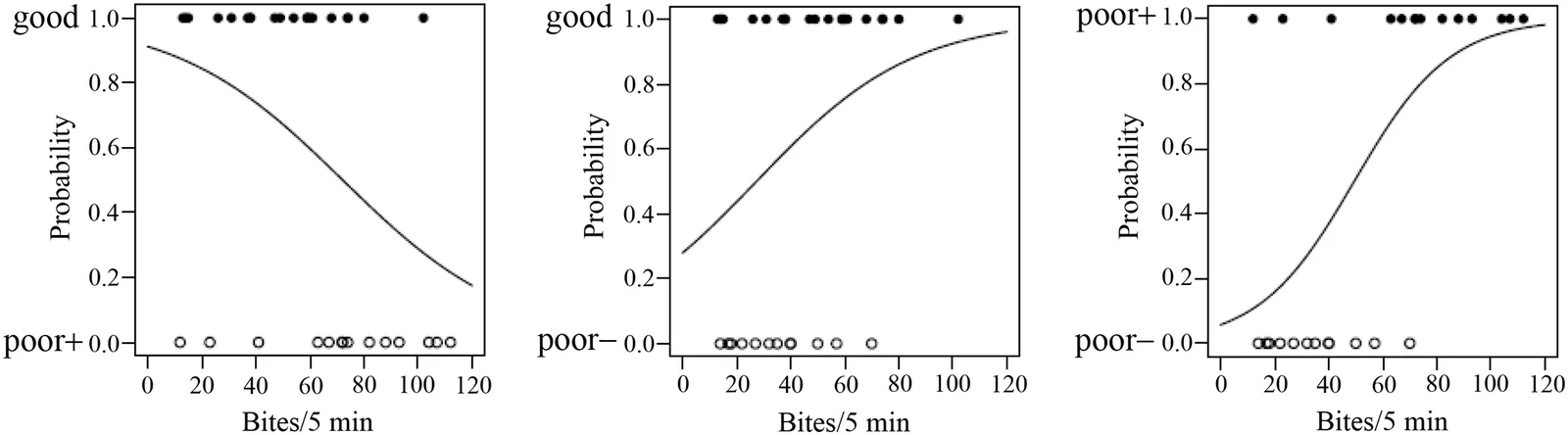
**Associations between intrinsic feeding responses to sucrose and learner phenotype probabilities in the DMSO-trained group.** Logistic regression analyses showing the associations between the intrinsic feeding response to sucrose, that is, the number of bites during 5 min at the pretest, and the estimated probabilities of each learner phenotype. The graphs compare the probabilities between (left) good and poor+ learner phenotypes, (middle) good and poor− learner phenotypes, and (right) poor+ and poor− learner phenotypes. The curves indicate the predicted probabilities derived from logistic regression models. The analyses were based on the same snails in the DMSO-trained group shown in Fig. 2 (good learners: *n*=18, poor+ learners: *n*=13, poor− learners: *n*=12). Each data point represents an individual snail (biological replicate).

Fig. 4 shows temporal changes in feeding responses during CTA training in each experimental group. Because of the time required to record feeding responses during CTA training, these responses were recorded only in a subset of randomly selected snails. Therefore, the following results are based on data obtained from those snails. The number of bites of each learner phenotype showed a marked decrease after the first conditioning trial, followed by a gradual reduction across subsequent conditioning trials. This temporal trend was observed in all experimental groups.

**Fig. 4.**
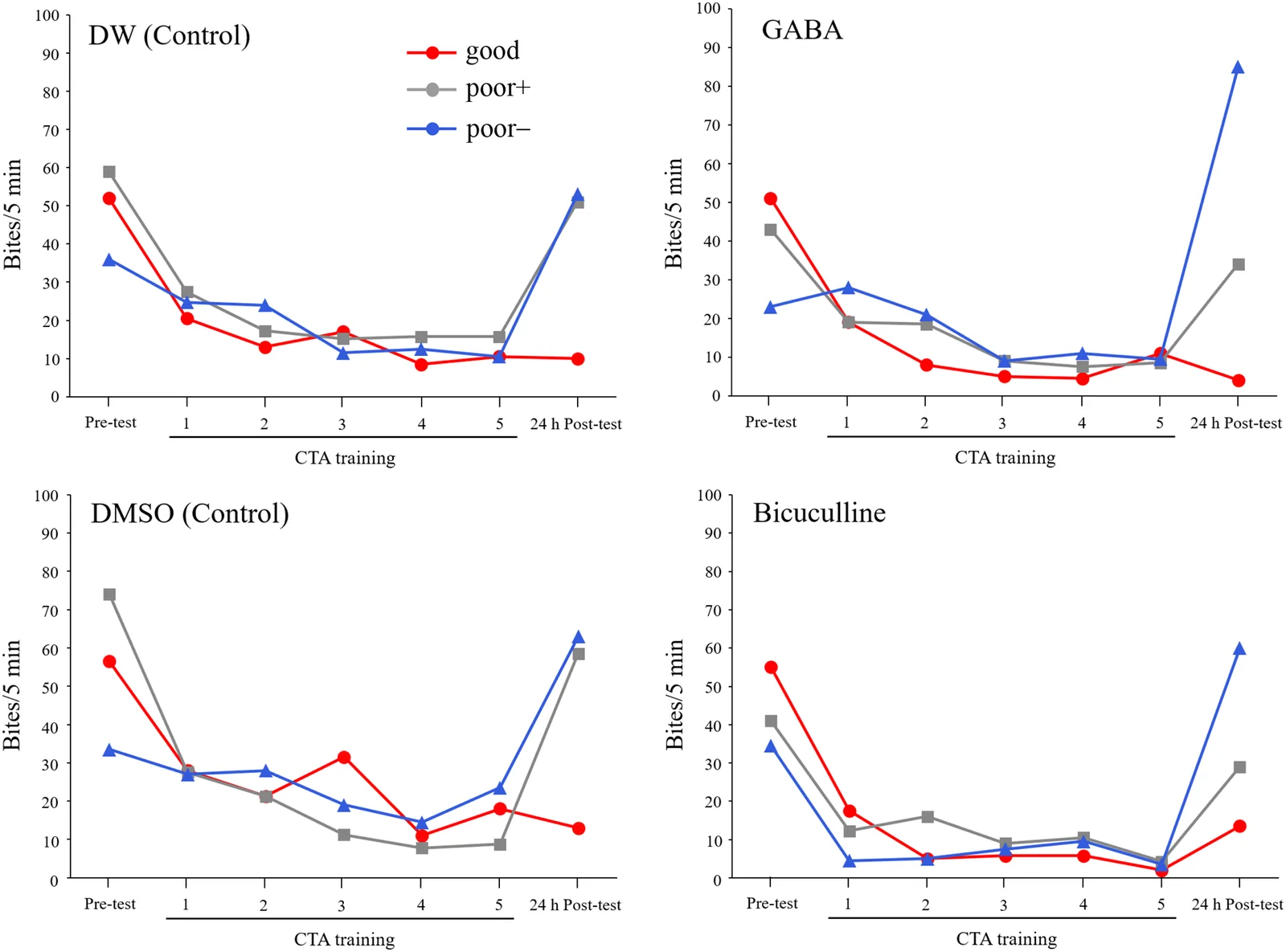
**Temporal changes in feeding responses during CTA training under GABAergic modulation.** Numbers of bites at the pretest, during CTA training, and at the 24 h posttest in the DW-, GABA-, DMSO- and bicuculline-trained groups. Red, gray and blue indicate good, poor+ and poor− learners, respectively. At the pretest and posttest, the number of bites was measured for 5 min and expressed as bites/5 min. During CTA training, the number of bites was measured for 10 min after each of the five conditioning trials and converted to average bites/5 min. The numbers 1-5 show the ordinal numbers of the five conditioning trials. The *n* values at the pretest and posttest were 80 in the DW-trained group, 23 in the GABA-trained group, 43 in the DMSO-trained group, and 41 in the bicuculline-trained group. Measurements during CTA training were performed for 53 out of 80 snails in the DW-trained group, 23 out of 23 snails in the GABA-trained group, 11 out of 43 snails in the DMSO-trained group, and 17 out of 41 snails in the bicuculline-trained group: *n*=23 for good learners, *n*=14 for poor+ learners and *n*=16 for poor− learners in the DW-trained group; *n*=9 for good learners, *n*=7 for poor+ learners and *n*=7 for poor− learners in the GABA-trained group; *n*=4 for good learners, *n*=4 for poor+ learners and *n*=3 for poor− learners in the DMSO-trained group; *n*=10 for good learners, *n*=4 for poor+ learners and *n*=3 for poor− learners in the bicuculline-trained group. Data are presented as medians; ranges are not shown to maintain clarity of the temporal trajectories. Each snail represents an independent biological replicate.

## DISCUSSION

In the present study, we analyzed behavioral differences among learner phenotypes and examined how pharmacological manipulation of GABAergic signaling during CTA training influenced learning performance and learner phenotype distribution. The principal findings were that learner phenotypes were associated with individual differences in intrinsic feeding responses to sucrose before training and that the GABA_A_ receptor antagonist bicuculline significantly increased the proportion of good learners. Such behavioral findings suggest that GABAergic inhibition is involved in regulating the feeding neural network. We therefore propose the working model illustrated in Fig. 5A. A previously proposed model (Kojima et al., 1997; Ito et al., 2012) is shown in black, whereas the red part represents a hypothetical component added in the present study and is described in detail below.

**Fig. 5.**
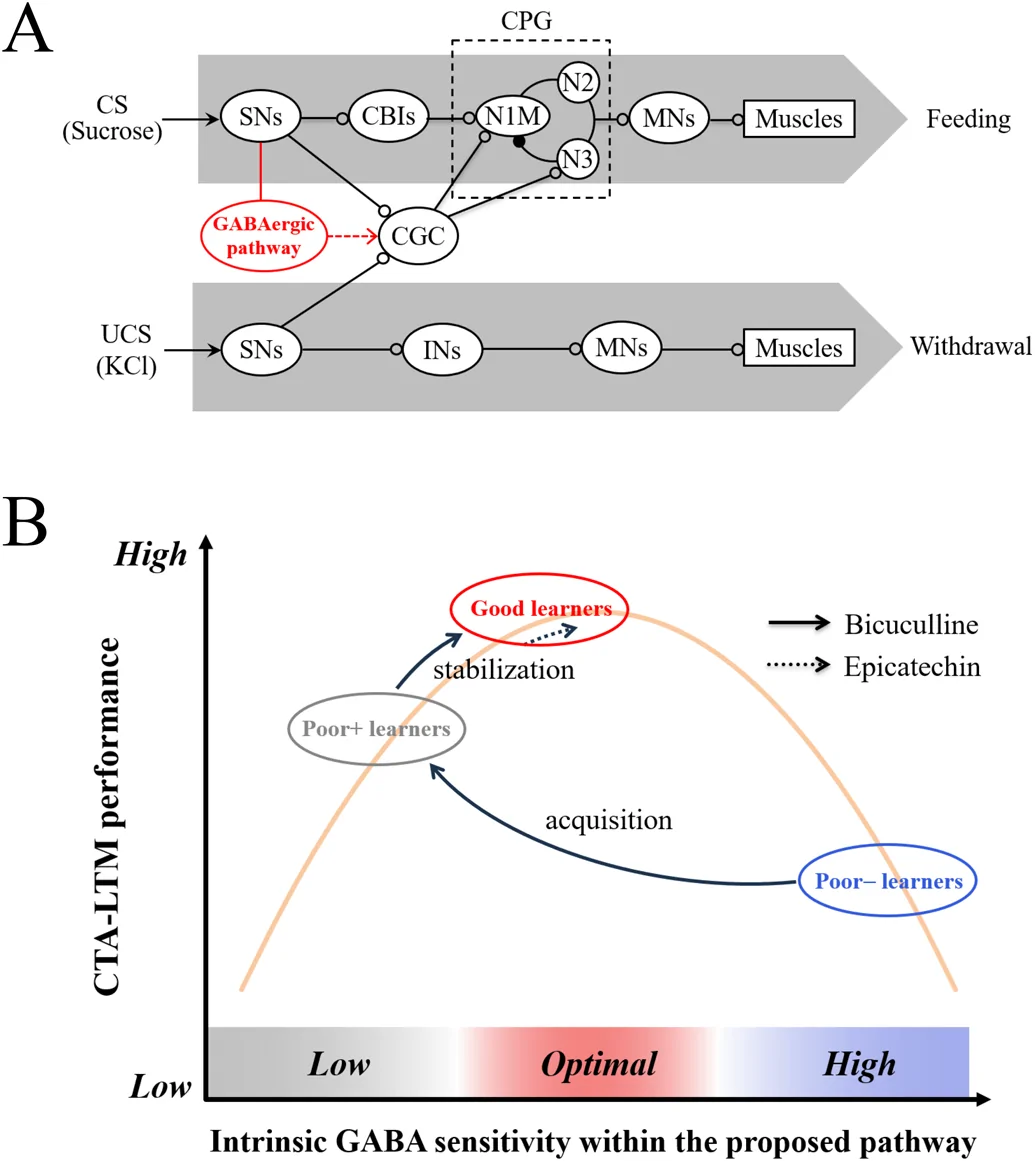
**Proposed feeding neural network model incorporating a putative GABAergic pathway and the proposed relationship between intrinsic GABA sensitivity and CTA-LTM performance.** (A) Schematic illustration of the proposed feeding neural network model. The black part is based on previous models, whereas the red part represents the putative GABAergic pathway proposed in the present study. The dashed arrow indicates the hypothetical inhibitory input to the CGC. Open and closed circles show excitatory and inhibitory inputs, respectively. CS: conditioned stimulus, UCS: unconditioned stimulus, SNs: chemosensory neurons, INs: interneurons, CBIs: cerebral buccal interneurons, CPG: central pattern generator, N1M, N2, N3: interneurons forming the CPG shown by a dashed square, MN: motor neurons. This figure is reproduced from Kojima et al. (1997), with permission. This image is not published under the terms of the CC-BY licence of this article. For permission to reuse, please see Kojima et al. (1997). (B) Schematic illustration of the proposed bell-shaped relationship between intrinsic GABA sensitivity within the proposed pathway and CTA-LTM performance. High or low intrinsic GABA sensitivity is proposed to impair CTA-LTM formation, whereas an intermediate level may provide optimal conditions for successful memory formation. The solid and dashed arrows indicate the proposed effects of bicuculline and epicatechin, respectively.

The previous feeding network model provides a useful framework for interpreting the present behavioral findings. The feeding CPG comprises several identified interneurons, including N1M, N2 and N3, in the buccal ganglia. Among these, the N3 cell suppresses the activity of the N1M cell (Benjamin and Elliott, 1989; Elliott and Kemenes, 1992; Yeoman et al., 1996). Sucrose is detected by chemosensory neurons (SNs) located in the lips and tentacles, and the sensory information is transmitted to the cerebral ganglia (Kemenes et al., 1986; Nakamura et al., 1999). These inputs activate both the CGC and the cerebral buccal interneurons (CBIs). Direct excitatory inputs from the CBIs activate the feeding CPG, generating the feeding motor output. The CGC modulates the excitability of the feeding CPG and regulates the initiation and maintenance of feeding motor patterns (McCrohan and Audesirk, 1987; Yeoman et al., 1996; Benjamin, 2012). KCl activates the CGC through sensory pathways distinct from those activated by sucrose (Nakamura et al., 1999). Activation of the CGC ultimately suppresses feeding behavior through modulation of the feeding CPG. During CTA training, the association between sucrose (CS) and KCl (UCS) is thought to occur in the CGC, inducing long-lasting suppression of feeding responses to sucrose (Kojima et al., 1997; Ito et al., 2012; Sunada et al., 2017a,b). After CTA training, activation of the CGC induces strengthened inhibitory transmission from the N3 cell to the N1M cell within the feeding CPG (Kojima et al., 1997) and induces plastic changes in the weak excitatory monosynaptic connection from the CGC to the N1M cell (McCrohan and Audesirk, 1987; Benjamin and Elliott, 1989).

Anatomical studies have shown that GABAergic neurons are widely distributed throughout the CNS of *Lymnaea* (Hatakeyama and Ito, 2000), and physiological studies have demonstrated that GABAergic transmission modulates the activity of feeding-related neurons (Elliott and Kemenes, 1992; Yeoman et al., 1996). In addition, our previous electrophysiological study showed that bath application of GABA to isolated CNS preparations suppressed spontaneous firing of the CGC after CTA-LTM formation (Itoh et al., 2021). These results led us to propose a working model incorporating a putative GABAergic pathway to the CGC, as illustrated by the red components in Fig. 5A. However, the proposed pathway remains to be demonstrated experimentally.

The proposed GABAergic pathway may be involved in CTA-LTM formation. The present study showed that the intrinsic feeding response to sucrose, that is, the initial (i.e. at the pretest) number of bites, was much lower in poor− learners than in good or poor+ learners (Figs 1E and 2E). In addition, the initial number of bites was slightly higher in poor+ learners than in good learners. These results may reflect individual differences in intrinsic GABAergic sensitivity within the proposed GABAergic pathway to the CGC. In poor− learners, strong GABAergic inhibition within this pathway may suppress CGC activation during sucrose presentation. Such excessive inhibition may prevent sufficient CS-UCS association during training. This interpretation is consistent with the present result that the proportion of good learners increased significantly when the GABA_A_ receptor antagonist bicuculline was applied after each conditioning trial (Fig. 2B).

This redistribution of learner phenotypes induced by bicuculline application can be interpreted as described in the Results section based on Fig. 3. Thus, individuals that would otherwise have been classified as poor− learners shifted to the poor+ learner category, whereas those that would otherwise have been classified as poor+ learners shifted to the good learner category. These stepwise transitions also suggest that GABA sensitivity within the proposed pathway may contribute to successful CTA-LTM formation. These proposed transitions, however, should be interpreted with caution because they are inferred from exploratory logistic regression analyses, and some learner subgroups, particularly the poor− subgroup in the bicuculline-trained group, contained relatively small numbers of snails.

Within this working framework, pharmacological reduction of GABAergic inhibition by bicuculline may reduce excessive inhibition in poor− learners with high intrinsic GABA sensitivity, allowing them to move from complete acquisition failure toward partial learning [black arrow (acquisition) in Fig. 5B]. In contrast, poor+ learners, which failed to show robust long-term suppression of feeding responses, exhibited a slightly higher initial number of bites compared to good learners. If the initial number of bites reflects intrinsic GABA sensitivity within the proposed pathway, intrinsic GABA sensitivity in poor+ learners may be slightly lower rather than higher. Therefore, their failure cannot be explained by excessive GABAergic inhibition, as proposed for poor− learners. Instead, when intrinsic GABA sensitivity is relatively low, learning-induced enhancement of GABA responsiveness may be insufficient to stabilize LTM. Thus, poor− and poor+ learners may fail to form CTA-LTM through distinct mechanisms.

Accordingly, successful CTA-LTM formation may require an optimal range of intrinsic GABA sensitivity (Fig. 5B). This idea is consistent with the possibility of an inverted-U relationship between intrinsic GABA sensitivity and memory outcome, although the present experiments did not directly test this relationship. We suppose that the proposed GABAergic pathway regulates the functional state of the CGC during training and that differences in intrinsic GABA sensitivity within this pathway may influence whether the CGC reaches the range required for successful CTA-LTM formation. Generally, inhibitory circuits have been shown to actively contribute to the formation and stabilization of memory representations rather than functioning solely as suppressive pathways (Caroni, 2015; Letzkus et al., 2015; Ferguson and Cardin, 2020). Therefore, one possibility is that bicuculline reorganizes the balance of excitatory and inhibitory inputs during training, rather than simply reducing inhibition, thereby enabling more effective stabilization of memory traces [black arrow (stabilization) in Fig. 5B].

As shown in Fig. 4, the number of bites of each learner phenotype showed a marked decrease after the first conditioning trial, followed by a gradual reduction across subsequent conditioning trials. These results suggest that the immediate suppression of feeding behavior induced by the aversive stimulus is relatively robust. Thus, pharmacological manipulation of GABAergic signaling after each conditioning trial had little apparent effect on the overall pattern of reduction in feeding responses during training. However, reduction of GABAergic inhibition by bicuculline appears to affect whether the accumulated experience reaches the level required for successful CTA-LTM formation (Fig. 2A,B).

In contrast to bicuculline, GABA applied after each conditioning trial did not significantly alter learning performance (Fig. 1A,B). This suggests that simply increasing GABAergic inhibition by GABA application after each conditioning trial is not sufficient to affect CTA-LTM formation. On the other hand, the differences in intrinsic feeding responses among learner phenotypes appeared to be reduced, and the significant differences between good and poor− learners and between poor+ and poor− learners observed in the control (DW-trained group) were no longer detected in the GABA-trained group (Fig. 1E,F). These changes may also reflect some redistribution among learner phenotypes, as suggested for the bicuculline experiment. In this experiment, however, the statistical power to detect such changes may have been limited because the number of snails in the GABA-trained group was relatively small.

By comparison, bath application of epicatechin to the CNS of naïve snails reduced the spontaneous firing rate of the CGC, indicating that epicatechin suppresses CGC excitability similarly to GABA (Itoh et al., 2021). Interestingly, epicatechin applied after each conditioning trial prolonged CTA-LTM persistence up to 72 h. However, the proportions of good, poor+ and poor− learners were similar to those in the control (DW-trained group). These findings suggest that epicatechin does not markedly influence the initial acquisition of CTA-LTM. Rather, within the framework of the present model, epicatechin may facilitate the learning-induced enhancement of GABA responsiveness within the proposed pathway, thereby promoting memory stabilization and prolonging CTA-LTM persistence (dashed black arrow in Fig. 5B).

The present interpretation assumes that the pharmacological agents reached neural circuits involved in CTA-LTM formation. Although the accessibility of externally applied agents to the CNS has not been directly demonstrated in *Lymnaea*, several observations suggest that pharmacologically active compounds can influence the CNS after external application. For example, epicatechin, a water-soluble compound, has been reported to cross the skin membrane of *Lymnaea* (Wertz, 2013), and the open circulatory system of gastropods may facilitate the distribution of externally applied agents. However, direct experimental verification of concentrations of these agents in the CNS will be required, especially for water-insoluble bicuculline. In addition, further pharmacological studies using a water-soluble GABA_A_ receptor antagonist, such as bicuculline salt or gabazine, will be required to confirm whether the observed behavioral effect is specifically mediated by GABA_A_ receptors.

Finally, the proportion of good learners in the DW-trained group was lower than that observed in our previous study (Itoh et al., 2021), in which it was comparable to that observed in the present bicuculline-trained group. One possible explanation is that intrinsic properties of the proposed GABAergic pathway differed among snail populations maintained in our laboratory. Recent studies have shown that different strains of *Lymnaea* exhibit different learning abilities (Sunada et al., 2017b; Rivi et al., 2025). Such variation may also occur within a laboratory colony maintained over multiple generations. However, environmental factors, such as housing conditions, food availability, and seasonal variation, may also contribute to the observed individual variability in feeding behavior and CTA-LTM formation.

In conclusion, the present study suggests that GABAergic inhibition within a proposed pathway to the CGC is associated with learning performance in CTA-LTM formation in *Lymnaea*. Individual differences in intrinsic feeding responses to sucrose were associated with differences in learner phenotype, indicating that variation in intrinsic GABA sensitivity within this pathway may influence learning performance. Bicuculline application increased the proportion of good learners, whereas GABA application did not significantly alter learner phenotype distribution. Together with previous results, the present behavioral findings support a working hypothesis in which intrinsic GABAergic sensitivity within the proposed pathway may influence CTA-LTM acquisition, whereas learning-induced enhancement of GABA responsiveness within this pathway contributes to memory stabilization. The proposed GABAergic pathway to the CGC remains to be demonstrated experimentally. Once the existence of this pathway has been established, further studies will be required to determine whether the pathway directly inhibits the CGC, whether the CGC expresses GABA receptors, or whether the pathway instead involves polysynaptic mechanisms. Nevertheless, the present working hypothesis provides a plausible framework for integrating both the present behavioral findings and previous results.

## MATERIALS AND METHODS

### Animals

*L. stagnalis* used in the present experiments were supplied by Professor Etsuro Ito of Waseda University, Tokyo, Japan. These snails were originally derived from the stocks maintained by Vrije University in Amsterdam. The snails are simultaneous hermaphrodites. They were all maintained in dechlorinated tap water under a 12:12 light-dark cycle at 20°C and fed *ad libitum* on *Brassica rapa* var. *perviridis* (a relative of bok choy) that is called komatsuna in Japan. Ethical approval was not required at Nihon University for experiments using *L. stagnalis*.

### CTA training

Adult snails were randomly selected and randomly allocated to experimental groups. The snails were deprived of food for 24 h prior to CTA training. The snails were then placed in a 90-mm-diameter polystyrene Petri dish and immersed in DW for 30 min to acclimate them to their new environment. Then, the snails were immersed in an appetitive solution (10 mM sucrose) for 15 s. Then, the sucrose solution was quickly aspirated and replaced with DW, and the number of bites was counted in DW for 5 min (pretest).

Ten minutes after the pretest, CTA training was performed. During this training, the snails were immersed in a 10 mM sucrose solution for 15 s (i.e. CS). Then, the sucrose solution was quickly aspirated, and the snails were immediately immersed in a 10 mM KCl solution (i.e. UCS) for 15 s. The concentrations of sucrose and KCl solutions were determined according to previous reports (Kojima et al., 1996; Nakai et al., 2020). Following this conditioning, the snails were immersed in DW for 10 min, and their numbers of bites were counted. This procedure was repeated five times. In another experiment, after each conditioning trial, instead of being immersed in DW (control), the snails were immersed in a GABA solution (1 mM) or an epicatechin solution (15 mg/l) for 10 min. In a separate experiment, the snails were immersed in a bicuculline solution (0.1 mM, an antagonist of the GABA_A_ receptor) for 10 min after each conditioning trial. Bicuculline was dissolved in a 0.1% DMSO solution because it is insoluble in DW, and the DMSO solution was therefore used as the control.

The concentrations of GABA (1 mM) and bicuculline (0.1 mM) were chosen based on previous electrophysiological studies of inhibitory synaptic transmission in neural circuits (Isaacson and Scanziani, 2011; Ferguson and Cardin, 2020). GABA applied externally in the submillimolar to millimolar range has been shown to reliably modulate neuronal activity, whereas bicuculline at approximately 0.1 mM effectively blocks GABA_A_ receptor-mediated synaptic transmission. The concentration of GABA is also consistent with that used in our previous study (Itoh et al., 2021). The concentration of epicatechin (15 mg/l) was chosen to provide a dose equivalent to that used in humans. Furthermore, at this concentration, epicatechin does not affect critical homeostatic behaviors in *Lymnaea* (Fruson et al., 2012).

After CTA training, the snails were kept in DW for 24 h, after which the posttest was performed. During the 24-h interval between the CTA training and the posttest, the snails were allowed only to feed *ad libitum* for the first 2 h to maintain their general physiological conditions. At the 24 h posttest, the number of bites was counted in DW for 5 min, as at the pretest.

We calculated the decrease in the number of bites at the 24 h posttest compared to that at the pretest as follows:

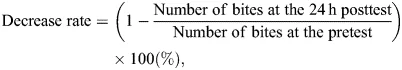


In our previous study, we categorized snails that showed a decrease rate of at least 40% as ‘good learners’, while those that showed a decrease rate of less than 40% were categorized as ‘poor learners’, so the ratio between good and poor learners could be about 2:1. Here, the poor learners include snails whose number of bites increased at the 24 h posttest; that is, the decrease rate of those snails was negative. In the present study, we further divided the poor learners into two groups, one of which showed a positive decreasing rate (0-40%) and the other showing a negative decreasing rate, for more detailed analyses. We here categorized the former snails as ‘poor+’ learners and the latter ones as ‘poor−’ learners.

### Statistical analysis

The proportions of learner phenotypes (good, poor+, and poor−) were compared between the following pairs of experimental groups using Fisher's exact test for 2×3 contingency tables: DW-trained group (control) versus GABA-trained group, DW-trained group (control) versus epicatechin-trained group, and DMSO-trained group (control) versus bicuculline-trained group. The *P*-values for the two comparisons involving the DW-trained group were adjusted for multiple comparisons using the Holm-Bonferroni method.

The initial (i.e. at the pretest) number of bites was compared among the three phenotypes within each experimental group. First, the normality of data distributions in each experimental group was assessed using the Shapiro-Wilk test. The results indicated that some data did not follow normal distributions. Therefore, nonparametric statistical methods were employed for all subsequent comparisons. The Kruskal-Wallis test was used to evaluate overall differences among the three phenotypes. Pairwise comparisons were then performed using Dunn's test, and the *P*-values were adjusted using the Holm-Bonferroni method.

Separate binary logistic regression analyses were performed for each pairwise comparison of learner phenotypes to investigate the relationship between the learner phenotype and the initial number of bites. The learner phenotype and the initial number of bites were used as the dependent and independent variables, respectively. The regression curves were generated using the glm function with a binomial distribution and a logit link.

All data were analyzed and plotted using R version 4.3.3 and GraphPad Prism 9. Unless otherwise indicated, data are presented as median and range. Differences were considered significant at *P*<0.05. Sample sizes were not determined by *a priori* power analysis. No animals were excluded from the analyses after the experiments were completed. The investigators were not blinded to treatment allocation during the experiments or outcome assessment.

### Use of artificial intelligence tools

ChatGPT (OpenAI) was used to assist with English-language editing and improvement of clarity. All AI-assisted text was reviewed and edited by the authors, who take full responsibility for the content of the manuscript.

## Supplementary Material

10.1242/biolopen.062762_sup1 Supplementary information
