# Supplementary material for: A proposed GABAergic pathway in the feeding neural network is associated with learning performance in conditioned taste aversion in Lymnaea
Source: Biol Open. 2026 Oct 16;15(10):bio062762. doi: 10.1242/bio.062762 (PMC13714873; doi:10.1242/bio.062762)
Supplement: Supplementary file 1 [file biolopen-15-062762-s1.pdf]

Epicatechin

A

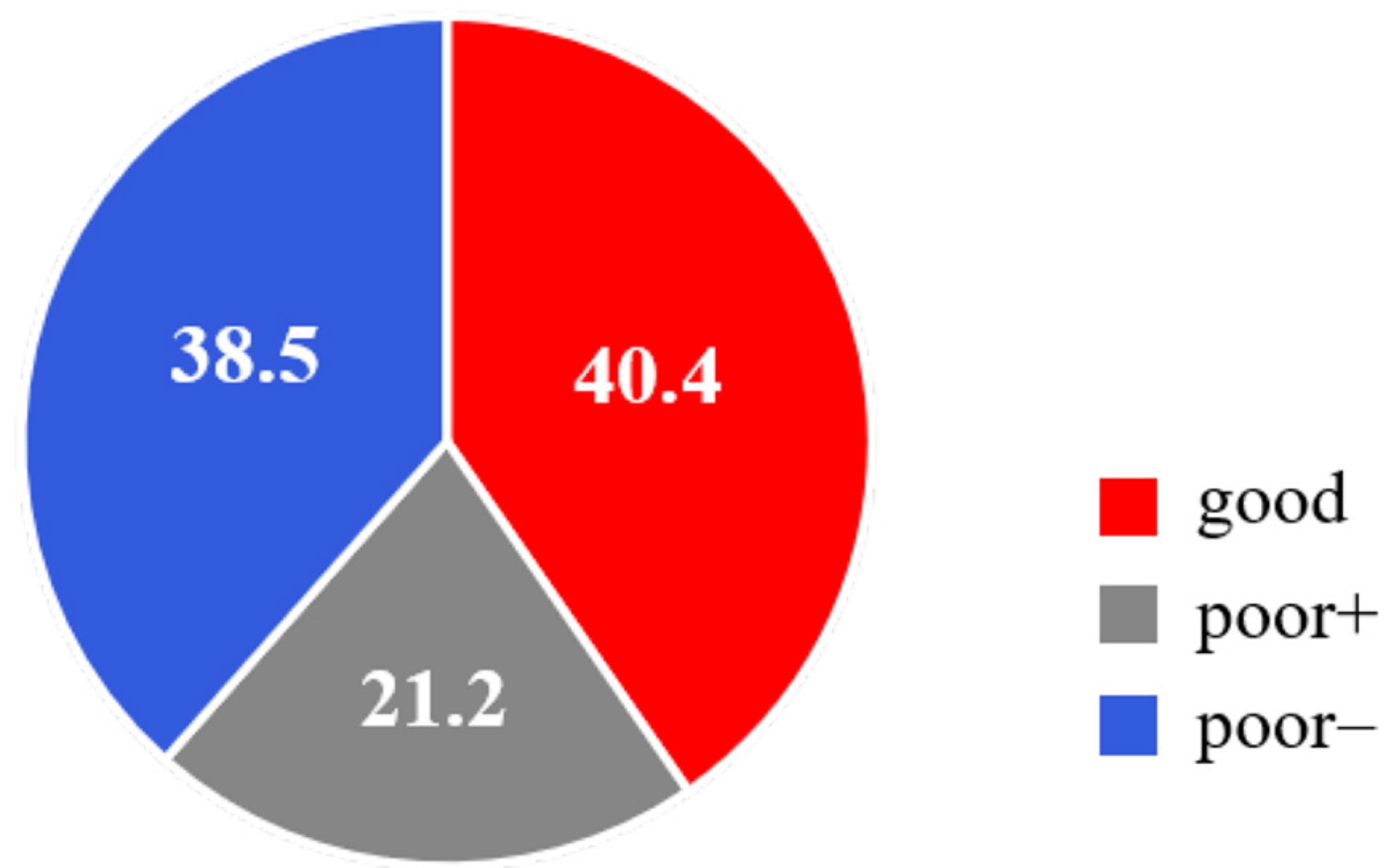

B

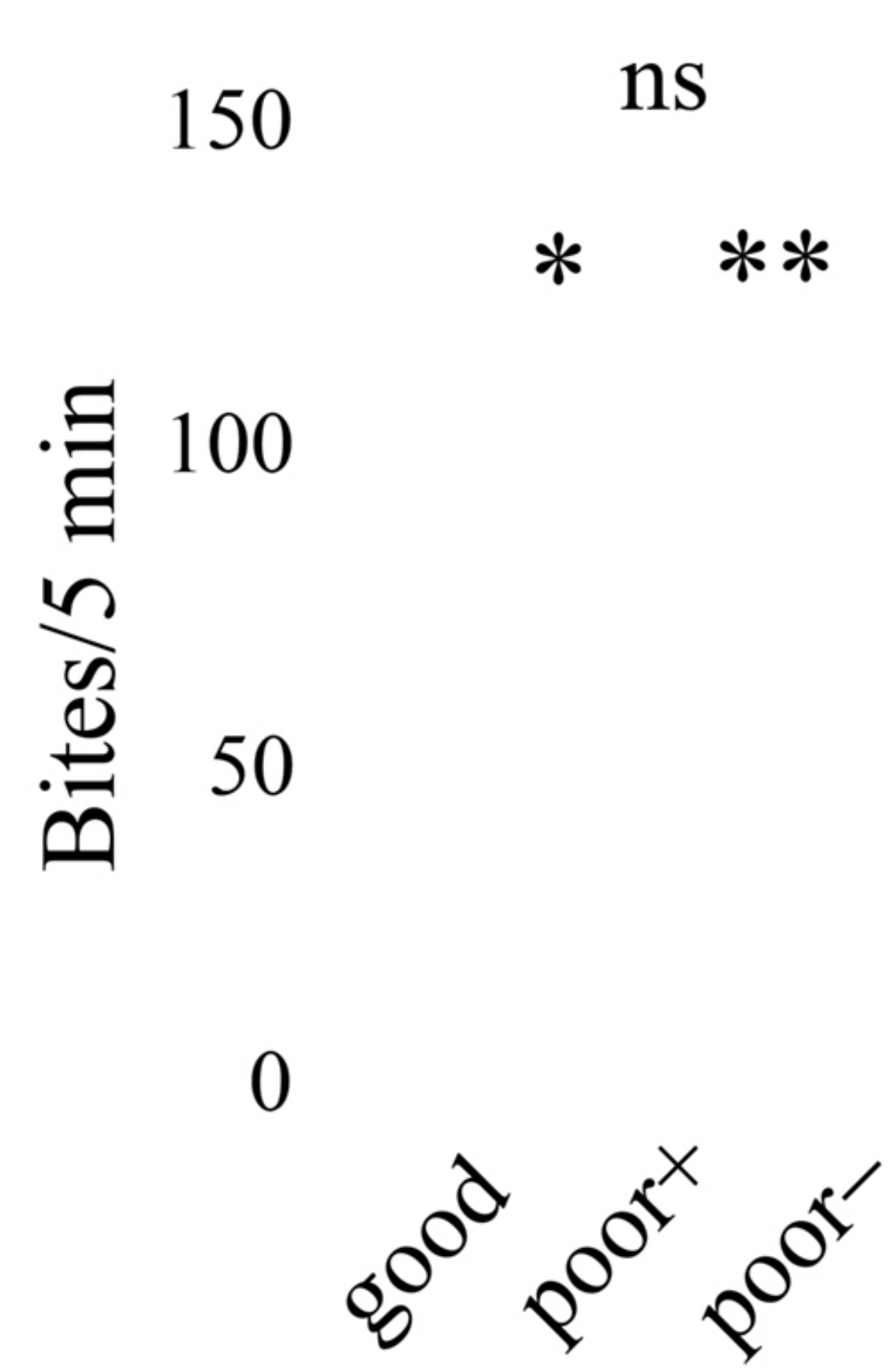

Biology Open • Supplementary information

Fig. S1. Effects of epicatechin treatment during training on learner phenotype distribution and intrinsic feeding responses to sucrose.

(A) Proportions of good, poor+ and poor− learners in the epicatechin-trained group (n=52). (B) Initial number of bites for 5 min at the pretest in the epicatechin-trained group (good learners: n=21, poor+ learners: n=11, poor− learners: n=20). Data are presented as individual values, medians and ranges (minimum–maximum). Data in B were compared using the Kruskal–Wallis test followed by Dunn's test with the Holm–Bonferroni correction for multiple comparisons. Where applicable, statistical tests were two sided. *P<0.05, **P<0.01, ns: not significant.

Biology Open • Supplementary information
